# *Beadex* Function in the Motor Neurons Is Essential for Female Reproduction in *Drosophila melanogaster*

**DOI:** 10.1371/journal.pone.0113003

**Published:** 2014-11-14

**Authors:** Subhash Kairamkonda, Upendra Nongthomba

**Affiliations:** Molecular Reproduction, Development and Genetics, Indian Institute of Science, Bangalore, Karnataka, India; Columbia University, United States of America

## Abstract

*Drosophila melanogaster* has served as an excellent model system for understanding the neuronal circuits and molecular mechanisms regulating complex behaviors. The Drosophila female reproductive circuits, in particular, are well studied and can be used as a tool to understand the role of novel genes in neuronal function in general and female reproduction in particular. In the present study, the role of *Beadex*, a transcription co-activator, in Drosophila female reproduction was assessed by generation of mutant and knock down studies. Null allele of *Beadex* was generated by transposase induced excision of P-element present within an intron of *Beadex* gene. The mutant showed highly compromised reproductive abilities as evaluated by reduced fecundity and fertility, abnormal oviposition and more importantly, the failure of sperm release from storage organs. However, no defect was found in the overall ovariole development. Tissue specific, targeted knock down of *Beadex* indicated that its function in neurons is important for efficient female reproduction, since its neuronal knock down led to compromised female reproductive abilities, similar to *Beadex* null females. Further, different neuronal class specific knock down studies revealed that *Beadex* function is required in motor neurons for normal fecundity and fertility of females. Thus, the present study attributes a novel and essential role for *Beadex* in female reproduction through neurons.

## Introduction

The nervous system plays an important role in modulating several physiological processes and complex behaviors in multicellular animals. Drosophila has served as an excellent model to unravel the neuronal regulation of multiple complex behaviors like memory and learning, aggression, courtship and female reproduction [1]–[3]. The neuronal regulation of female reproduction in particular has been studied extensively and the multiple circuits which play a major role have been identified. Several studies have shown that octopaminergic neurons from the central nervous system regulate multiple female reproductive behaviors like ovulation, egg laying and also sperm release [4]–[8]. An e*x vivo* study has demonstrated the direct role of octopamine in the contraction of the Drosophila female reproductive tract [9]. Glutamatergic neurons also modulate the contraction of oviduct by acting in conjunction with octopamine during egg laying [10]. A recent study has revealed that octopamine brings about the contraction/relaxation of oviduct through CamKII mediated signaling in the epithelial cells of oviduct [7]. Apart from neuronal circuits intrinsic to the female, seminal proteins transferred from the male during copulation are also essential for inducing post mating behaviors in female through these neuronal circuits [5]. For example, Sex peptides (SP) transferred from male into the female reproductive tract during copulation bind to receptors on the sensory neurons of the female reproductive tract, that project on to the central nervous system regions (known to send projections on to female reproductive tract), and bring about post mating reproductive behaviors like increased ovulation, egg laying and reduced receptivity [11], [12]. With such well studied neuronal circuits regulating the female reproduction and simple reproductive assays, the Drosophila female reproductive system serves as a good model to understand the functions of uncharacterized genes implicated in neuronal functions.

*Beadex* (*Bx*) was isolated as a spontaneous mutant with beaded wings (long, narrow and excised along both margins) in Drosophila (http://flybase.org, version 13 FB2008_09) [13]. An elegant study by Lifschytz and Green showed that the mutant wings' phenotype was caused by over production of the *Bx* gene product (hypermorph) [14]. Zhu and co-workers in 1995 identified a homolog of human LIM domain oncogene in Drosophila [15]. Later three independent studies reported that *Bx* itself codes for Drosophila LIM only (dLMO) domain containing protein [16]–[18]. Any disruption in the *Bx* coding sequence leads to *Bx* loss of function and is evident as held-up wings phenotype. However, abrogation in the 3′UTR sequence, which harbors negative regulatory elements, leads to gain of function alleles of *Bx* [17].

The LIM domain consists of two tandemly organized zinc finger motifs with the conserved cysteine-histidine residues that co-ordinate zinc binding. LIM domain mediates its function through protein-protein interactions [19]. In *Bx* hypermorphs, increased Bx levels disrupt the functional complex of Apterous-Chip (Ap-Chip) due to the competitive binding of Bx to LIM-binding domain (LBD) of Chip [18], [20]. Bx also plays an important role in the development of dorsal macrochaetae (bristles on the dorsal side of thorax) by activating the transcription of *Acheate* and *Scute* through its positive interaction with Pannier (Pnr) in the sensory organ precursor (SOP) specification [21], [22]. *Lmo4*, one of the vertebrate homologues of *Bx*, suppresses the formation of excitatory glutamatergic inter-neurons and promotes the development of GABAergic inter-neurons in mice [23]. Apart from regulating the above mentioned developmental processes, *Bx* also affects Drosophila behavior in response to cocaine and ethanol [24]–[26]. Bx affects ethanol mediated sedation through repression of *anaplastic lymphoma kinase* (*Alk*) in the brain of Drosophila [25]. The modulation of activity of *Bx* homologues in mice, *Lmo4* and *Lmo3*, also affect the behavior of mice in response to cocaine and ethanol, through mechanisms similar to those in Drosophila [26], [27].

Though several studies have reported *Bx* role in multiple behaviors through neurons, the molecular mechanisms and the targets through which *Bx* effects its function are not clearly understood. In this study, we attribute a novel and essential function for *Bx* in Drosophila female reproduction. We show that multiple, post mating reproductive processes like ovulation, egg laying and sperm release are compromised in the *Bx* null females. We further provide evidence that *Bx* regulates these functions through the motor neurons that innervate the female reproductive tracts.

## Materials and Methods

### Fly stocks and Genetics

All the flies were maintained at 23±2°C, under 12 hrs Light/Dark (L/D) cycles on cornmeal-sucrose-yeast agar media. Either *w^1118^* females or female progeny of Canton-S males crossed with *w^1118^* females were used as controls, unless specified. *Bx^hpd-G14-1^* was a kind gift from Pascal Heitzler (CNRS, France, described in [21]). New *Bx* alleles were generated by mobilizing *P{GawB}Bx^hdp-G14-1^* element using *P{Δ2–3}* as source for transposase (Bloomington Stock (BS)#4368) [28]. Hop-outs from F1 were balanced over FM7a followed by screening of F2 progenies for white eye (excision of P-element results in white eye). These white eyed females were crossed with wild type males and resulting F3 males were screened for held-up wings (since, as reported earlier, *Bx* loss of function alleles show held-up wings phenotype) [17]. Isolated hop-out alleles which showed held-up wings were maintained over duplication of *Bx* locus on Y chromosome, *Dp(1;Y)W39* [29]. *Bx* RNAi line was obtained from Vienna Drosophila RNAi Centre (VDRC Transformant ID#2917). All the Gal4 lines and GFP tagged reporter protein construct fly lines were procured either from Bloomington Drosophila stock centre, Indiana or National Center for Biological Sciences (NCBS) stock centre, Bangalore, India.

### Molecular characterization of *Bx^7^* allele

**Figure 1:**
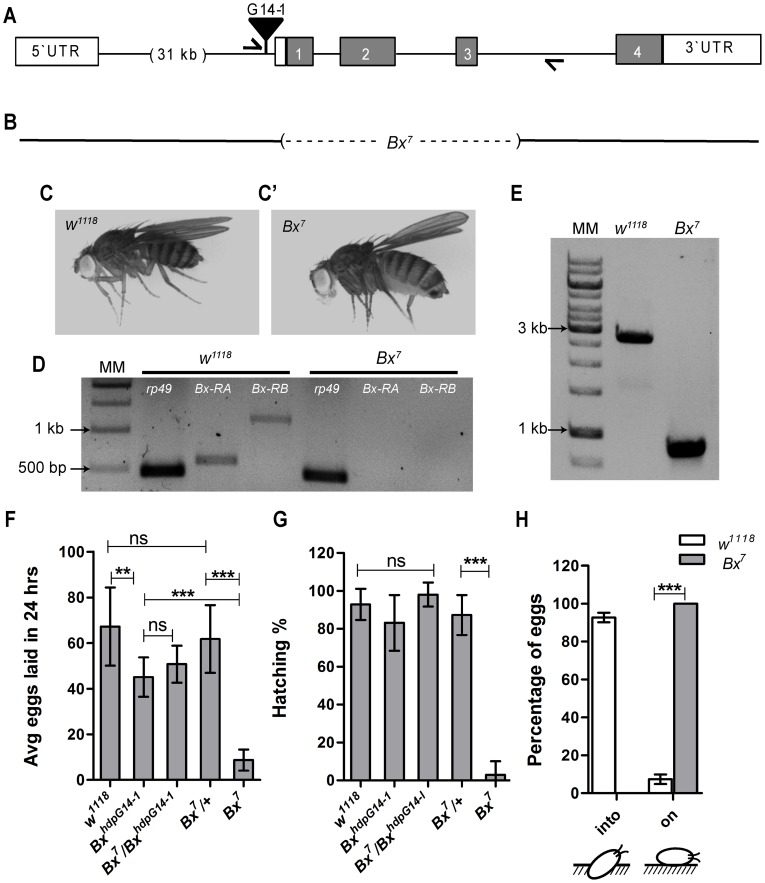
*Bx* null females show compromised reproduction. *Bx* null allele was generated for assessing their reproductive ability. (A) Genomic location of G14-1 P[GawB] element which was mobilized using Δ2–3 transposase. (C, C') *Bx* null flies (*Bx^7^*) generated showed held-up wings. (D) RT-PCR analysis showed complete absence of *Bx*-RA and *Bx*-RB transcript products. (E) PCR amplification of genomic DNA of *Bx^7^* flies showed deletion of ∼2 kb from *Bx* gene sequence. Position of primers used for the genomic PCR is depicted in A and genomic region deleted in *Bx^7^* is represented in B. (E) *Bx^7^* females have highly reduced fecundity (F, ***, p<0.0001; **, p = 0.0011) and fertility (G, ***, p<0.0001) (no of eggs tested>800 (*w^1118^* and controls), ∼200 (*Bx^7^*)) (Students unpaired t-test with Welch's correction) compared to wild type and other controls. (H) almost 100% of the eggs are laid on the surface of the media by *Bx^7^* mutant females compared to control females which deposit upto 90% of the eggs into the media (schematic of eggs laid by control and *Bx^7^* mutant females is shown below the X-axis of the graph) (***, p<0.001, Two way anova with Bonferroni post tests).

RNA from whole body of 1–2 days old *w^1118^* and *Bx* hop-out flies was isolated using trizol (Sigma) following manufacturer's instructions. From the RNA, 2 µg was taken for making cDNA using first strand cDNA kit following manufacturer's protocol (Thermo Scientific, India). Primers specific for *Bx*-RA and *Bx*-RB transcript, were designed as follows- *Bx*-RAFP 5′ – CTAATTGAGTCGAGTGTGCGTG -3′, *Bx*-RARP 5′ – AAGGAGGTTGGTTGTCGTCGTC -3′, *Bx*-RBFP 5′ – ATGGAGTACCTCTACAACGCTA – 3′ and *Bx*-RBFP 5′ – TTATTTCGGGACCCGTAC – 3′. House-keeping gene *rp49* was used as control using following primers *rp49*FP 5′ – TTCTACCAGCTTCAAGATGAC – 3′ and *rp49*RP 5′ – GTGTATTCCGACCACGTTACA – 3′. In order to characterize the molecular lesion in hop-out *Bx* allele, genomic DNA was isolated from wild type (*w^1118^*) and isolated *Bx* mutant (*Bx^7^*). Primers were designed on either side of the p{GawB} *Bx^hdp-G14-1^* insertion site (assessed from [21]). The sequences of primers used for PCR amplification of *Bx* gene region are *Bx*-FP 5′ – GGCTCGTTGGTCTAGAGGTA – 3′ and *Bx*-RP 5′ – CATAATGGCATCTCCGCAAG – 3′. The location of the primers in the *Bx* gene is represented in Figure 1A. PCR amplification of *Bx* gene region was performed with ExTaq (TaKaRa). The resultant PCR products were analyzed through agarose gel electrophoresis and documented using Alpha DigiDoc RT2 (JH BIO Innovations, India). Further, the PCR product from the *Bx* mutant was cloned into TA vector (InsTAclone PCR cloning kit, Thermo scientific, India) and sequenced (Amnion, India). The resultant sequences were BLAST aligned against Drosophila genome (http://flybase.org/blast/) to determine the molecular lesion in the *Bx* mutant.

### Gal4 expression profiling

In order to check the expression pattern of neuronal Gal4 lines in the female reproductive system, females of w^*^, Elav-Gal4; +; + (BS#458, expresses Gal4 pan-neuronally, [30] Lin and Goodman 1994), w^*^; dTdc2-Gal4; + (BS#9313, expresses Gal4 in the pattern of the tyrosine decarboxylase 2 gene involved in octopamine synthesis, [31] and w^*^, VGlut-Gal4; +; + (BS#24635, expresses Gal4 in many, but not all glutamatergic neurons, personal communication of Saxton to Flybase, 2007.12.13) were crossed with w^*^, UAS-SyteGFP; +; + (BS#6924) males. For *Dmef2*-Gal4 (BS#27390, expresses Gal4 in muscle cells) [32] expression profile, Gal4 females were crossed with mCD8-GFP (BS#32186) males. All the progenies were grown at 29°C and the reproductive tract of 4–5 days old adult female progeny was dissected. The dissected tissues were fixed in 4% Paraformaldehyde in PBS (4% PF) and washed for 15 minutes in PBS containing 0.3% Triton-X (PBTx) (Sigma). Washed tissue samples were stained with phalloidin-TRITC (Sigma) for visualizing reproductive tract muscles. Tissue samples were washed again for 15 minutes (3×5 minutes) with PBTx and mounted in the Vectashield mounting media (Vector labs). Imaging was performed using Zeiss LSM510 Meta confocal microscopy and the images were processed with LSM software (version 3.2.0.115, Zeiss).

### Knock down studies

For efficient knock down studies, UAS-*Bx^RNAi^* construct was brought together with UAS-*Dcr2* (BS#24646) [33]. Similarly, to perform developmental stage specific knock down, tub-*Gal80^ts^* (drives temperature sensitive inhibitor of Gal4 activity, Gal80^ts^, under *alpha*-*tubulin84B* promoter, [34]) was brought along with UAS-*Bx^RNAi^*. Virgin females of *UAS-Dcr2*; *tub-Gal80^ts^*; *UAS-Bx^RNAi^* or *UAS-Dcr2*; +; *UAS-Bx^RNAi^* were crossed either with different Gal4 construct males or with wild type males (for heterozygote controls) and grown at appropriate temperatures (for knock down with *Dmef2-Gal4*, *Bx^RNAi^* without UAS-*Dcr2* was used, since knock down along with *Dcr2* caused larval lethality). For knock down across all the stages, progeny were grown from the early embryonic stage up to the test age (3–4 days old adult flies) at 29°C. For the pupal stage knock down, larvae were grown till wandering third instar stage at 18°C and were then shifted to 29°C till the adult flies eclosed. The eclosed flies were then kept at 18°C for the next 4–5 days. For the adult stage knock down of *Bx*, larvae and pupae were allowed to develop at 18°C and the freshly eclosed adult flies were kept at 29°C for next the 4–5 days.

### Reproductive ability assays – Fertility, Fecundity, Ovulation and Oviposition

Virgin females (3–4 days old) of interested genotype and 2–3 days old Canton-S males (grown at 25°C) were isolated for crosses. Single pair matings were set up in the media vials. After the visual confirmation of mating and recording the duration of copulation, males were separated and the females were transferred into egg laying media vials (1% Sucrose, 1% Agar-Agar and 0.2% Propionic acid) supplemented with yeast paste and kept in 25°C or 29°C incubator with 12 hrs L/D cycle. The females were removed 24 hrs later and the number of eggs laid was counted to assess the fecundity. Fecundity was plotted as the average number of eggs laid per female in 24 hrs. Further, same set of vials were incubated at 25°C and checked 24 hrs later to count the hatched eggs for the fertility assay. Fertility was plotted as percent hatched eggs. For assessing the ovulation ability of the females, 24 hrs post mating (hpm) with Canton-S males, reproductive tract was dissected and the percent of females showing the presence of egg in the uterus was recorded. Minimum of 15 females per genotype were used for all the experiments and the experiments were independently repeated at least three times. For oviposition assay, mated females were transferred on to petri plates with egg laying media and kept at 25°C or 29°C incubator for 6 hrs. The females were then removed and the percent of total eggs deposited on the media was recorded. Data for all the above assays were plotted as mean with standard deviation.

### Ovariole development

Ovaries of the females mated with wild type males were dissected and the number of developing ovarioles was counted in each ovary. Following this, the same samples were fixed in 4% PF for 30 minutes and washed for 15 minutes (3×5 minutes) with PBTx. The washed samples were then incubated with Hoechst (1 µg/ml, Sigma) for 30 minutes for nuclear staining, washed again in PBTx and mounted in Vectashield mounting media (Vector labs). The mounted samples were visualized and imaged using Zeiss fluorescent microscope (Zeiss Axio Observer Z1).

### Sperm storage assay

To study the sperm storage and releasing ability, females of the interested genotype were mated with 2–3 days old *dj*-GFP males, which have GFP labeled sperm tails (BS#5417) [35]. At 6 hpm and 10 days post mating (dpm), the female reproductive tract was dissected, fixed in 4% PF and washed for 15 minutes in PBTx. Following this, the samples were stained with phalloidin-TRITC (Sigma) for visualizing seminal receptacle. The presence or absence of sperms was monitored by looking for GFP signal in the seminal receptacle. Imaging was performed using Zeiss LSM510 Meta confocal microscopy and the images were processed with LSM software (version 3.2.0.115, Zeiss).

### Data plotting and Statistical analysis

Data was plotted, graphs were made and statistical test analysis was performed using Graph pad Prism 5 (Graphpad).

## Results

### Generation of *Bx* hypomorphic alleles

LIM domain of Bx can bind to proteins containing LBD and can thus regulate the function of several proteins. In order to study whether *Bx* regulates the female reproduction or not, generation of loss of function or hypomorphic alleles of *Bx* was essential. New *Bx* alleles were generated by mobilizing *P{GawB}* element (*Bx^hdp-G14^* ^-*1*^) inserted near 3′ end of the first intron (Figure 1A, and described in [21]). Out of several individual hop-outs obtained, twelve lines were revertants and three showed held-up wings phenotype. Of these three, only *Bx^7^*, which showed severe held-up wings defect, was characterized and used in the present study (Figure 1C & C'). cDNA prepared from the *Bx^7^* mutant flies revealed complete absence of transcript products of *Bx*-RA and *Bx*-RB isoforms (Figure 1D). Furthermore, PCR amplification of genomic DNA of the hop-out revealed deletion of ∼2 kb of *Bx* gene sequence (Figure 1E) which harbors a *Bx*-RB specific exon (1), two constitutive exons (2 & 3) and about 70 bp of the 5′UTR (Figure 1B). Thus, *Bx^7^* is a null allele of the *Bx*.

### *Bx* null females show reduced fecundity and fertility

In order to find whether *Bx* regulates the female reproduction, *Bx^7^* females were mated with Canton-S males. *Bx^7^* females showed normal receptivity (100% receptivity- data not shown) and normal mating duration (Figure S1). However, despite showing normal mating duration, mutant females showed highly reduced fecundity and fertility (egg hatching percentage) compared to wild type (*w^1118^*) and other controls (*Bx^7^*/+, *Bx^hdp^* ^-*G14-1*^ */Bx^hdp^* ^-*G14-1*^, and *Bx^7^*/*Bx^hdp^* ^-*G14-1*^) (Figure 1F and 1G). It is interesting to note that homozygous *Bx^hdp-G14^* ^-1^ also showed reduced fecundity compared to controls. This indicated that insertion of *P{GawB}* element also affects the *Bx* function. The unhatched eggs were white in color but not brown indicating a fertilization defect. Another evident phenotype of the *Bx^7^* females was abnormal oviposition, wherein all the eggs laid by the mutant females were deposited on the surface of the media in contrast to the control females which deposited eggs into the media (Figure 1H).

### *Bx* null females show normal ovary development but defective post mating responses

**Figure 2:**
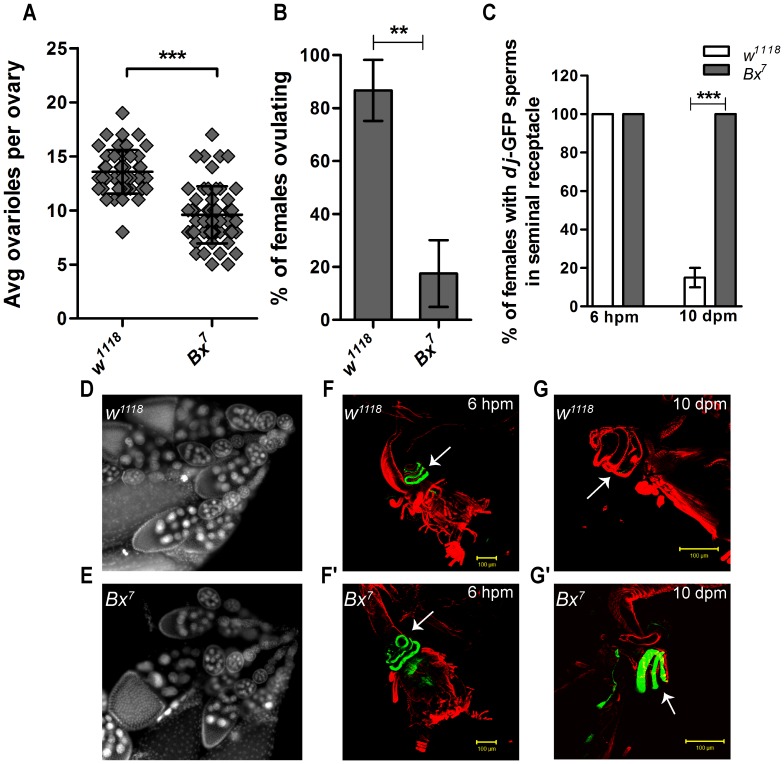
*Bx^7^* mutant females show normal ovariole development but reduced post mating responses. (A, B) *Bx^7^* females have reduced number of ovarioles (unpaired t-test, ***, p<0.0001) and also reduced ovulation (unpaired t-test, **, p = 0.0017). (C) Sperm storing and releasing ability of the controls and the *Bx^7^* mutant females at 6 hpm and 10 dpm respectively (unpaired t-test, ***, p<0.0001). (D, E) Ovarioles stained for nucleus with Hoechst showed normal developmental stages in the control (D) and *Bx^7^* (E) females. (F, F') *Bx^7^* and the control females, after mating with *dj*-GFP males, store sperms in the seminal receptacle within 6 hpm. (G, G') Control flies emptied all the sperms in the seminal receptacle by 10 dpm (G) while *Bx^7^* females failed to release sperm from the seminal receptacle even after 10 dpm (G'). Green-*dj*-GFP sperms, Red-Phalloidin-TRITC.

Ovaries of *Bx^7^* and control females were analyzed to see if the reduced fecundity was due to abnormal ovary development. At gross level, it could be observed that the number of ovarioles was less in the *Bx^7^* mutant females compared to those in the control females (Figure 2A). Nuclear staining of *Bx^7^* ovarioles with Hoechst showed normal development of the ovarioles across various stages similar to that of the controls, indicating normal oogenesis in *Bx^7^* mutant females (Figure 2D and 2E). Mating induces several physiological and behavioral changes in the females which include increased egg laying (fecundity), ovulation and oogenesis [36]–[38]. Since the mutant females showed reduced fecundity, we checked if the ovulation was defective in the mutant females. Indeed, the *Bx^7^* mutant females showed highly reduced ovulation compared to the controls (Figure 2B), indicating that the reduced fecundity could be the result of reduced ovulation rather than a defect in the development of ovarioles. However, we can't completely rule out developmental role of *Bx* in ovaries during ovariole development, as ovariole numbers have been shown to be influenced by cell differentiation and numbers established during metamorphosis stage [39]. For an efficient post mating response, sperms and the accessory gland proteins bound to sperms must be stored in the female sperm storage organs- spermathecae and seminal receptacle [40], [41]. Moreover, only 10% of the eggs laid by the mutant females hatched (Figure 1G). So we looked at the sperm storing ability of the mutant females and the control females by crossing them with the *dj*-GFP males. As shown in the Figure 2F and F', within 6 hpm *dj*-GFP labeled sperms accumulated in seminal receptacle in both the control (*w^1118^*) and *Bx^7^* mutant females. Thus, *Bx^7^* mutant females have normal sperm storing ability. Over a period of 10–15 dpm, sperms stored in the storage organs are released for fertilizing eggs, thus depleting sperms from the storage organs [42]. As seen in Figure 2G, *dj*-GFP sperms from the seminal receptacle of the control flies (*w^1118^*) were totally depleted by 10 dpm. However, the seminal receptacle of *Bx^7^* mutant females retained *dj*-GFP sperms even after 10 dpm (Figure 2C and 2G'). This could be one possible reason for the reduced hatching of the eggs laid by the mutant females.

### *Bx* function in the neurons, but not in the muscles, is required for the female reproductive success

**Figure 3:**
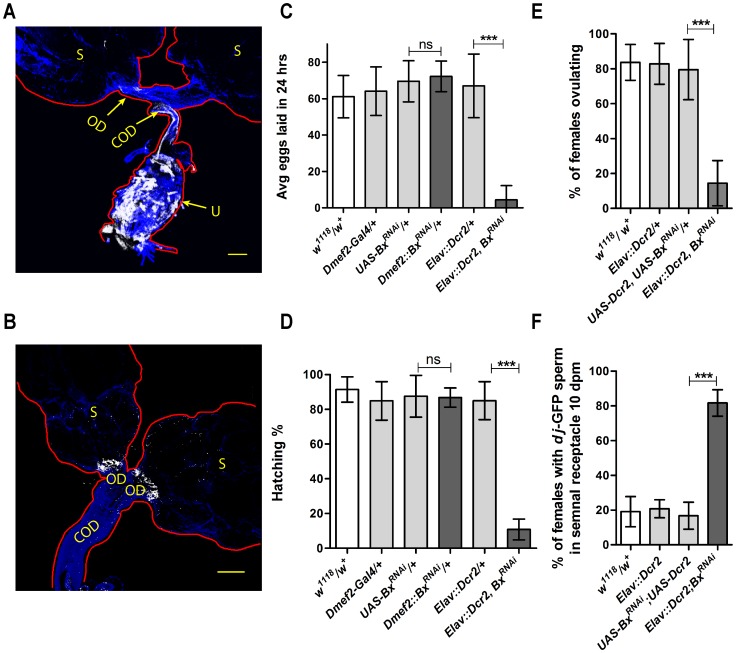
*Bx* function in the neurons is essential for the normal female reproduction. In order to test in which of the tissues was *Bx* essential for the female reproduction *Bx* was knocked down in the muscles (*Dmef2*-Gal4) and neurons (*Elav*-Gal4) individually. (A) *Dmef2*-Gal4 driven expression of mCD8GFP showed its expression in the muscles of uterus (U), common oviduct (COD) and oviduct (OD) (blue-Phalloidin-TRITC, white-mCD8GFP). (B) *Elav*-Gal4 driving UAS-*Syt*eGFP (Green) expression showed innervations of the neurons onto the female reproductive system muscles which include OD, COD and peritoneal sheath (S) (also see Figure S2). (C, D) Knock down of *Bx* in the neurons but not in the muscles reduces fecundity (C) and fertility (D) (C & D- unpaired t-test with Welch's correction, ***, p<0.0001) (no of eggs tested >1000 (*w^1118^* and controls), ∼200 (*Bx^7^*)). (E, F) Knock down of *Bx* in the neurons also reduces ovulation (E) and sperm release (F) in females (one way anova, with Dunnetts' post tests. E- ***, p = 0.0005, F-***, p<0.0001).

A detailed analysis of multiple reproductive behaviors of the *Bx^7^* null females indicated that the defective phenotypes could result from reduced motor activity of the reproductive tract. Motor activity of the reproductive tract is modulated through the muscles and neurons. In order to test which of the tissues was affected in the *Bx^7^* mutant females, *Bx* was knocked down either in the muscles or neurons separately and the flies were assessed for their fecundity and fertility. When *Bx* was knocked down in the ovarian muscles using *Dmef2*-Gal4 (which showed strong expression in all the female reproductive tract muscles) (Figure 3A), neither fecundity nor fertility of the females was affected (Figure 3C and 3D). *Elav*-Gal4, a pan neuronal Gal4, showed strong expression in neurons innervating more or less all the muscles of the female reproductive tract (Figure 3B and Figure S2). Knock down of *Bx* in the neurons with *Elav*-Gal4 led to highly reduced fecundity and fertility which was comparable to the *Bx^7^* mutant females (Figure 3C and 3D). Even in this case the unhatched eggs were white in color, clearly indicating a defect in the fertilization of the eggs. Knock down of *Bx* in the neurons not only affected fecundity and fertility, but also phenocopied all the reproductive defects observed in the *Bx^7^* mutant females like reduced ovulation (Figure 3E), reduced sperm release from the seminal receptacle (Figure 3F) and defective egg deposition (Figure S3) with the exception that unlike the *Bx^7^* mutant females, the neuronal knock down of *Bx* led to more accumulation of mature eggs in the ovary (Figure S4).

### Developmental and post developmental function of *Bx* is essential for the female reproductive ability

**Figure 4:**
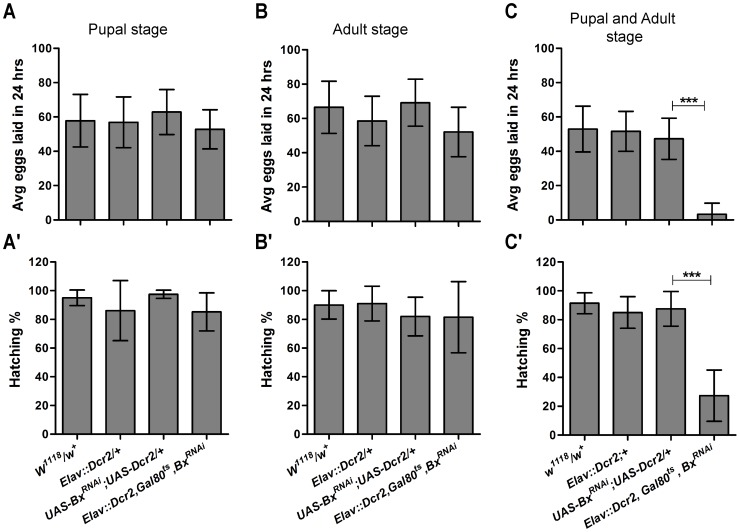
*Bx* function in the neurons is essential during both the pupal development and adult stage for efficient female reproduction. (A–B') Knock down of *Bx* in the neurons during pupal stages (A, A') or adult stage (B, B') did not affect either fecundity (A, B) or fertility (A', B') of the females. (C, C') Knock down of *Bx* in both the pupal and adult stages reduced fecundity (C) and fertility (C') significantly (unpaired t-test, ***, p<0.0001).

*Bx* acts as a transcription co-activator in several developmental processes like wing disc morphogenesis and bristle development [18], [20]–[22]. In order to understand if *Bx* function in the neurons was required during development for female reproduction, stage specific knock down of *Bx* in the neurons was carried out. *Bx* knock down during the pupal development or in the adult stage separately did not cause any reproductive defects (Figure 4A, A' and 4B, B'). However, knock down of *Bx* during both pupal and adult stages together showed significantly reduced fecundity and fertility of the females (Figure 4C, C'). It is possible that *Bx* loss of function during the pupal stage and associated developmental defects are compensated by the *Bx* expressed during the adult stage. However, such compensation would not happen with the adult stage specific knock down of *Bx*. Thus, it is possible that *Bx* function in the neurons is more critical during development of the female reproductive tract.

### *Bx* knock down in glutamatergic, but not aminergic neurons, partially affects the female reproductive abilities

**Figure 5:**
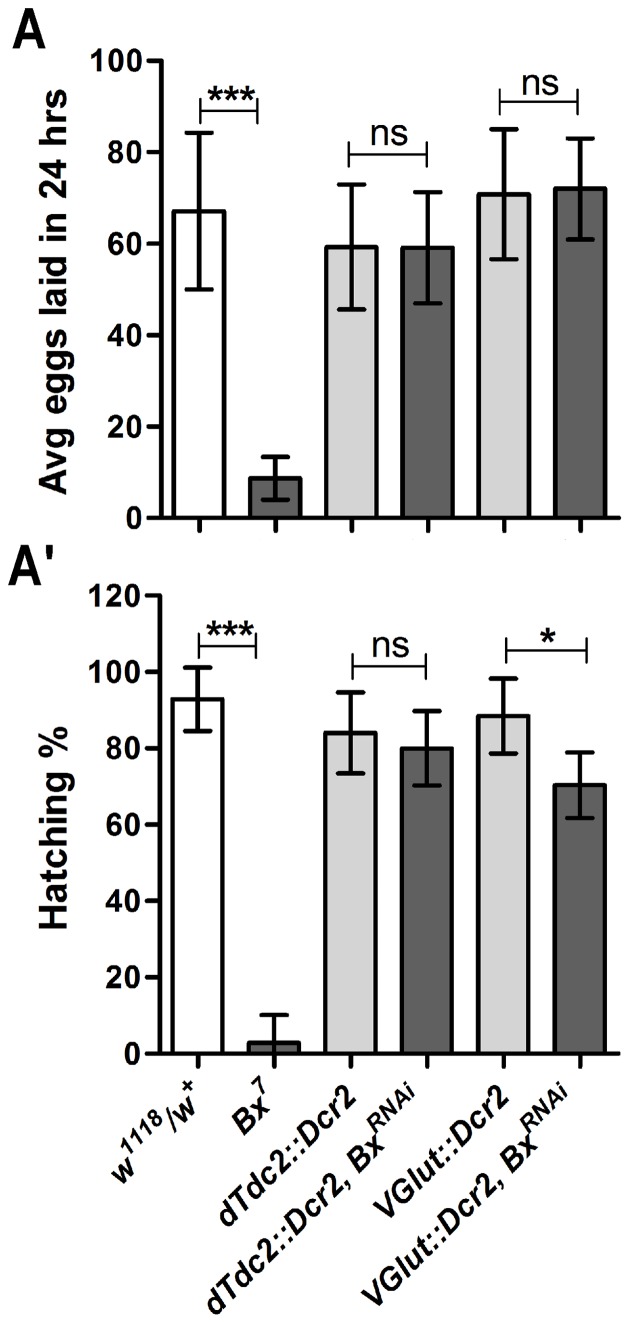
Knock down of *Bx* in the glutamatergic neurons but not in the octopaminergic neurons partially reduces female fertility. Knock down of *Bx* was performed in the octopaminergic neurons (*dTdc2*-Gal4) and glutamatergic neurons (*VGlut*-Gal4) separately and assessed for their fecundity (A) and fertility (A'). Knock down of *Bx* in octopaminergic neurons did not affect the female fecundity or fertility (A, A') (no of eggs tested ∼500 in control and test). Knock down of *Bx* in the glutamatergic neurons did not affect the female fecundity (A) but partially reduced fertility (A') (unpaired t-test,*, p = 0.0135, ***, p<0.0001) (no of eggs tested ∼800 in control and test).

Several genetic studies have revealed that octopaminergic and glutamatergic classes of neurons play critical roles in female reproductive behaviors like ovulation, fecundity and sperm storage [4]–[8]. Moreover, these classes of neurons show innervations onto various parts of the female reproductive tract [4], [10] (Figure S5). Thus, to find if *Bx* modulated the female reproduction through these classes of neurons, *Bx* was knocked down in octopaminergic and glutamatergic neurons using *dTdc2*-Gal4 and *VGlut*-Gal4 respectively. *dTdc2*-Gal4 neurons showed innervations onto the oviduct and uterine muscles of the female reproductive tract (Figure S5 C, D), while *VGlut*-Gal4 neurons showed projections only onto the uterine muscles (Figure S5 A, B). Knock down of *Bx* in the octopaminergic neurons did not affect either fecundity or fertility of the females (Figure 5A and 5B). However, females in which *Bx* was knocked down in the glutamatergic neurons showed a partial reduction in fertility without affecting fecundity (Figure 5B). Moreover, knock down of *Bx* in the glutamatergic neurons led to oviposition defect in the females similar to that of *Bx^7^* mutant females, though to a lesser extent (Figure S6). One of the possible reasons why knock down of *Bx* either in the octopaminergic neurons or glutamatergic neurons separately did not significantly affect female reproduction could be due to the innervation of these classes of neurons onto fewer female reproductive tract muscles.

### *Bx* function in the motor neurons is important for female reproduction

**Figure 6:**
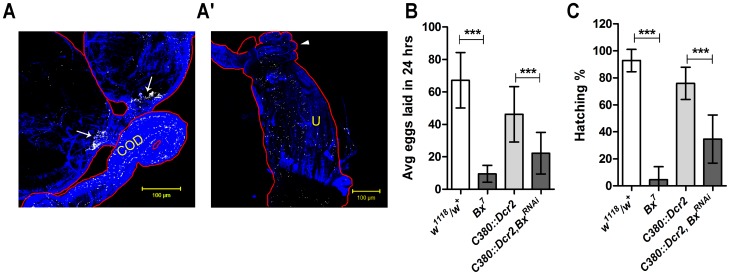
*Bx* function in the motor neurons is important for the female reproduction. (A, A') C380-Gal4 neurons driving expression of *Syt*-eGFP showed their projections on to most of the female reproductive tract muscles like oviduct (white arrows), common oviduct (COD), seminal receptacle (white arrow head) and uterus (U). Knock down of *Bx* in C380 motor neurons significantly reduced the female fecundity (B) and fertility (C) which can be compared to that of the *Bx^7^* mutant females (unpaired t test, ***, p<0.0001). Blue indicates Phalloidin-TRITC and white indicates *syt*-eGFP.

As mentioned earlier, the reproductive defects in *Bx^7^* mutants could be due to reduced motor activity caused by the abnormal neuronal function. In order to test this hypothesis, *Bx* was knocked down in the motor neurons innervating the female reproductive tract muscles using motor neuron specific Gal4 line C380 [43]. UAS-*Syt*eGFP reporter under C380-Gal4 revealed extensive innervations of this class of neurons onto the oviduct, common oviduct, uterine and seminal receptacle muscles (Figure 6A and 6B). Knock down of *Bx* in these motor neurons in the females resulted in significant reduction in fecundity (Figure 6C) and fertility (Figure 6D) compared to the controls. Moreover, the egg deposition defect was also recapitulated when *Bx* was knocked down in the C380 motor neurons (data not shown). This indicated that *Bx* function in the motor neurons is essential for female reproduction.

## Discussion

In this study, we report a novel and essential role of the *Bx* in Drosophila female reproduction. The *Bx* null females generated in this work showed normal ovariole development but with highly reduced fecundity and ovulation. Reduced fecundity correlates well with reduced ovulation. However, unlike earlier reported mutants which showed reduced fecundity and ovulation [4], accumulation of mature eggs in the ovaries is not seen in the *Bx^7^* mutant females. One possible reason for this could be the highly reduced abdominal cavity caused by the distended crop (Figure S7). This is supported by a recent study which identified *Bx* in a screen for defective gastric emptying, thereby causing bulged crop [44] (unpublished data from lab). Other reason could be due to the function of *Bx* in the ovariole development, which was not investigated in this study. Highly reduced fertility of *Bx^7^* mutants could be attributed to the failure of sperm release from the storage organs (seminal receptacle) during ovulation (Figure 2G'). There is no direct evidence as to what stimulates the release of sperms from the storage organs in the females. One of the proposed stimuli is egg movement/presence in the uterus which activates stretch receptors in the uterus and induces release of sperm from storage organs [42]. It is thus possible that the reduced sperm release observed in the *Bx^7^* null females is due to reduced ovulation.

The overall defects like reduced fecundity, sperm release from storage organs and abnormal oviposition (Figure 1 and 2) could be attributed to the reduced motor activity of the female reproductive tract. Motor activity of the female reproductive tract is brought about by neuromuscular activities. Neuronal knock down of *Bx* in the females phenocopied all the reproductive defects of the *Bx* null females (Figure 3). This clearly indicates that reproductive defects observed in the *Bx^7^* mutant are due to loss of *Bx* function in the neurons. However, it needs to be noted that the neuronal knock down of *Bx* leads to accumulation of mature eggs in the ovary unlike *Bx* null (Figure S4). This could be because there is no crop distension in flies with *Bx* knocked down in the neurons unlike the *Bx^7^* mutant females (data not shown). In the females where *Bx* is knocked down neuronally, it is also possible that *Bx* functions in the ovarioles are unaltered, which might lead to accumulation of mature eggs in the ovarioles.

*Bx* plays an important role during the development of wing disc and SOP [18], [20]–[22]. Similarly our study indicates that, for an efficient female reproduction, *Bx* function is essential during the development of the reproductive tract (pupal stage). During development, Bx might transcriptionally regulate the genes which affect the neuronal circuits innervating the female reproductive tract. However, Bx might work independent of Pnr, a known transcription factor that interacts with Bx, in regulating the female reproduction, since *pnr* knock down in the neurons does not affect fecundity or fertility (data not shown). *Lmo4*, a vertebrate homolog of *Bx* is known to affect the number of neurite outgrowths and their length in human SH-SY5Y neuroblastoma cells [45]. Similar to *Lmo4*, *Bx* also might regulate the projection of neurons on to target muscles during development. Though the present study does not show any evidence for such functions, it offers a look at the neuro-circuits regulating reproduction for dissecting the molecular mechanism of *Bx* function.

Octopaminergic circuits innervating the female reproductive tract are major players regulating fecundity, ovulation and sperm release [4]–[8]. However, knock down of *Bx* in these circuits did not cause any reproductive defects (Figure 5). One of the possible reasons could be that *Bx* might work through other circuits independently or in unison with the octopaminergic neurons for modulating female reproduction. For instance, glutamatergic neurons also play a vital role in oviduct contraction along with the octopaminergic neurons [10]. But, knock down of *Bx* in the glutamatergic neurons did not reduce fecundity, but reduced fertility only partially, and caused abnormal egg deposition on the surface of the media. This could be due to the strong innervations of glutamatergic neurons onto the uterine muscle (Figure 5, S5 and S6). Since *Bx* knock down in the octopaminergic neurons did not cause any defect and knock down in the glutamatergic neurons only gave a partial defect, it can be speculated that *Bx* functions through multiple class of neurons for regulating female reproduction. *Bx* regulates the development of peripheral sensory organs (dorsal macrochaete) [21], [22]. Female reproductive tract harbors sensory neurons which are stimulated by sex peptides from male ejaculum [11], [12]. To see if *Bx* affects the female reproduction through these sensory neurons, *Bx* was knocked down in these sensory neurons using *ppk*-Gal4 (BS#32078, expresses Gal4 in class IV dendritic arborization neurons and also in sensory neurons in female reproductive tract) [12]. However, *Bx* knock down in these sensory neurons did not cause any defect in fecundity or fertility (Figure S8).

Reproductive defects in the *Bx* null females might be caused by the reduced motor activity of the reproductive tract. This is supported by the fact that knock down of *Bx* in the motor neurons reduced fecundity and fertility of females significantly. This clearly shows that *Bx* activity in the motor neurons is essential for the female reproductive behavior. One possible explanation could be that *Bx* affects the neurotransmission activity thereby causing reduced female reproductive tract contractions. However, this may not be the case since *Bx* knock down in the neurons only in adult stage did not cause any reproductive defects in the females (Figure 4B). Drosophila Islet, a LIM-homeo domain protein, is essential in deciding the neurotransmitter identity and axon path finding in the motor neurons during embryonic development [46]. More recent studies also report the modulation of voltage-dependent fast K^+^ channel (encoded by *shaker*, *sh*) expression levels by Islet along with Lim3, another LIM-homeo domain protein [47], [48]. It is possible that in the *Bx^7^* mutants and in the neuronal knock down of *Bx*, the functions of Islet and Lim3 are modulated (either increased or decreased activity) in the neurons due to their reduced interaction with Bx and this might result in altered neuronal function and the described phenotypes. This further emphasizes that *Bx* function is crucial during the female reproductive tract development. Drosophila Protein Interaction Map (DPiM) project has identified almost 100 proteins which interact with Bx in S2 cell lines [49]. Of these only 18 proteins show high expression in the adult brains (Flybase, version FB2014_04). The function of these interacting partners could be affected in the neurons of *Bx^7^* mutant flies and also in the neurons with *Bx* knock down, there by affecting the female reproduction.

## Conclusion

We conclude that *Bx* is essential for Drosophila female reproduction where it regulates ovulation, sperm release from the storage organs and oviposition. All the reproductive defects of *Bx* null females can be phenocopied by knocking down *Bx* in the neurons. More specifically, *Bx* plays an important role in female reproduction through the motor neurons. This work shows the importance of utilizing the female reproductive tract circuits in understanding the molecular pathways through which *Bx* might modulate neuronal functions.

## Supporting Information

Figure S1 **Copulation duration of** ***Bx^7^*** **females is similar to that of wild type females.** Both wild type and *Bx^7^* mutant females showed normal duration of copulation when mated with wild type Canton-S males.
(TIF)

Figure S2 **Innervations of** ***Elav*** **-Gal4 neurons onto the female reproductive tract muscles.** *Elav*-Gal4 neurons showed innervations onto almost all the musculature of female reproductive tract like oviduct (OD), ovary sheath (S) and uterus (U). Blue- Phallodin-TRITC and Green-Syt-eGFP.
(TIF)

Figure S3 ***Bx*** **knock down in the neurons show oviposition defect.** Knock down of *Bx* in the neurons leads to defective oviposition where in close to 100% of the eggs was deposited on the surface of the media.
(TIF)

Figure S4 **Mature eggs accumulation in** ***Bx^7^*** **and the neuronal knock down of** ***Bx*** **females.** *Bx^7^* females do not show accumulation of mature eggs in the ovaries unlike those of control ovaries (*w^1118^*). However, knock down of *Bx* in the neurons in females leads to accumulation of mature eggs in the ovaries unlike controls.
(TIF)

Figure S5 **Innervation of** ***VGlut*** **-Gal4 and** ***dTdc2*** **-Gal4 classes of neurons onto the female reproductive tract muscles.** *VGlut* Gal4 class of neurons showed projections only onto uterine musculature (B, U) but not to the oviduct or common oviduct (A, OD). *dTdc2*-Gal4 class of neurons showed projections onto the oviduct (C, OD) and uterine musculature (D, U). Blue-Phalloidin-TRITC and Green-Syt-eGFP.
(TIF)

Figure S6 **Knock down of** ***Bx*** **in the glutamatergic neurons affects oviposition.** Knock down of *Bx* in the glutamatergic neurons leads to oviposition defect similar to *Bx^7^* mutant females, though to a smaller percentage. (unpaired t-test, ***, p<0.0001).
(TIF)

Figure S7 **Crop distension in** ***Bx^7^*** **mutant females reduces abdominal cavity space.** *Bx^7^* mutant females showed increase in the abdominal size due to distension of crop caused due to reduced food movement along the gastric tract. This might lead to inhibition of mature egg accumulation in the *Bx* mutant ovaries. Red arrow indicates bulged abdomen and crop.
(TIF)

Figure S8 ***Bx*** **does not regulate female reproduction through the sensory neurons in the female reproductive tract.** *Bx* was knocked down in the sensory neurons in the female reproductive tract with *ppk*-Gal4. However, this does not reduce either fecundity (A) or fertility (B) of the females.
(TIF)
