# Supplementary figures and images for: Beadex Function in the Motor Neurons Is Essential for Female Reproduction in Drosophila melanogaster
Source: PLoS One. 2014 Nov 14;9(11):e113003. doi: 10.1371/journal.pone.0113003 (PMC4232528; doi:10.1371/journal.pone.0113003)

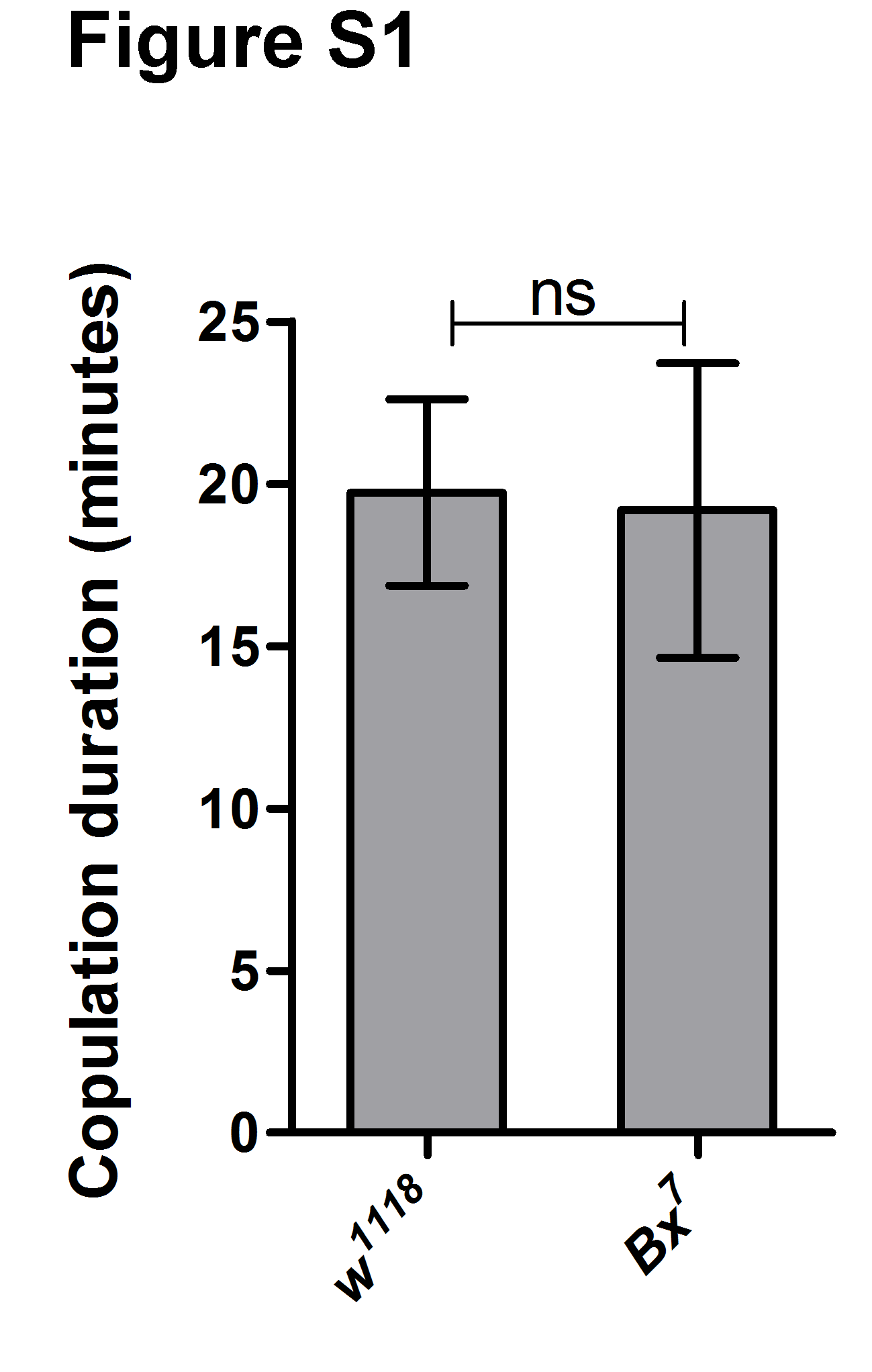

Supplement: Figure S1 — Copulation duration of Bx7 females is similar to that of wild type females. Both wild type and Bx7 mutant females showed normal duration of copulation when mated with wild type Canton-S males. (TIF) [file pone.0113003.s001.tif]

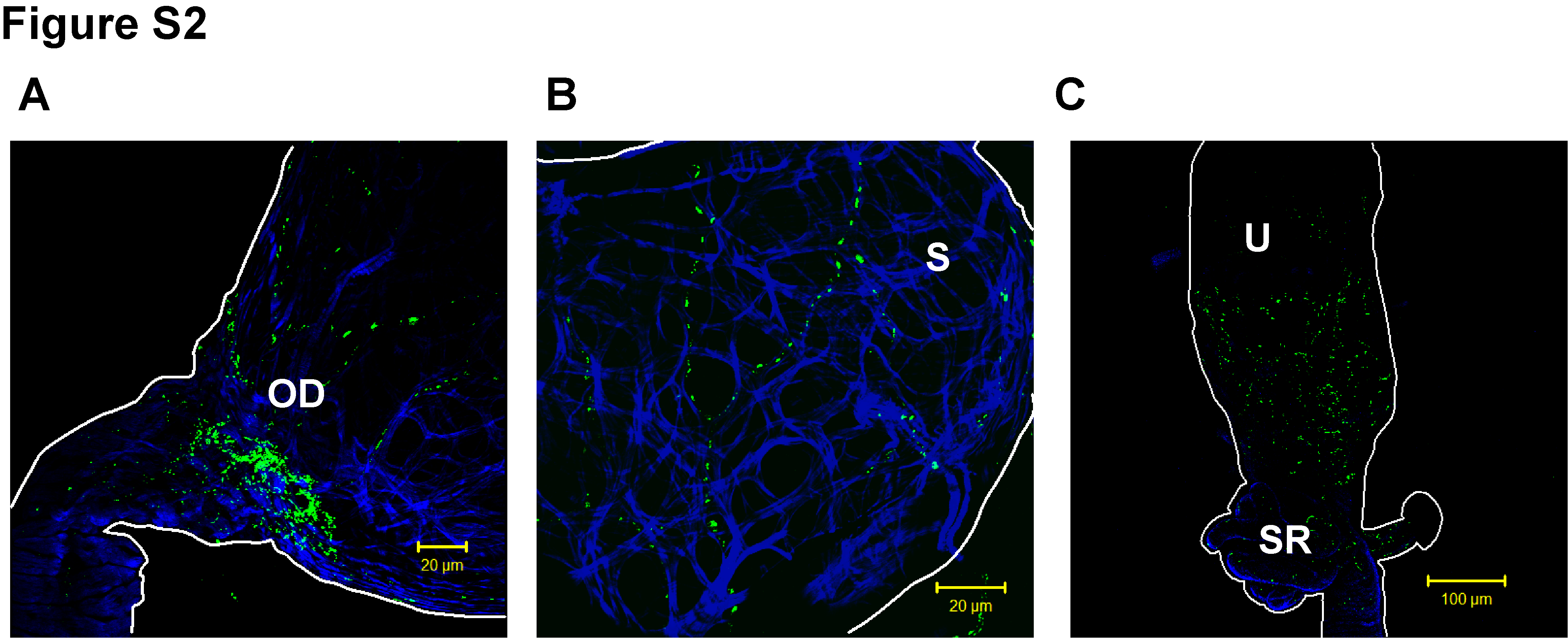

Supplement: Figure S2 — Innervations of Elav -Gal4 neurons onto the female reproductive tract muscles. Elav-Gal4 neurons showed innervations onto almost all the musculature of female reproductive tract like oviduct (OD), ovary sheath (S) and uterus (U). Blue- Phallodin-TRITC and Green-Syt-eGFP. (TIF) [file pone.0113003.s002.tif]

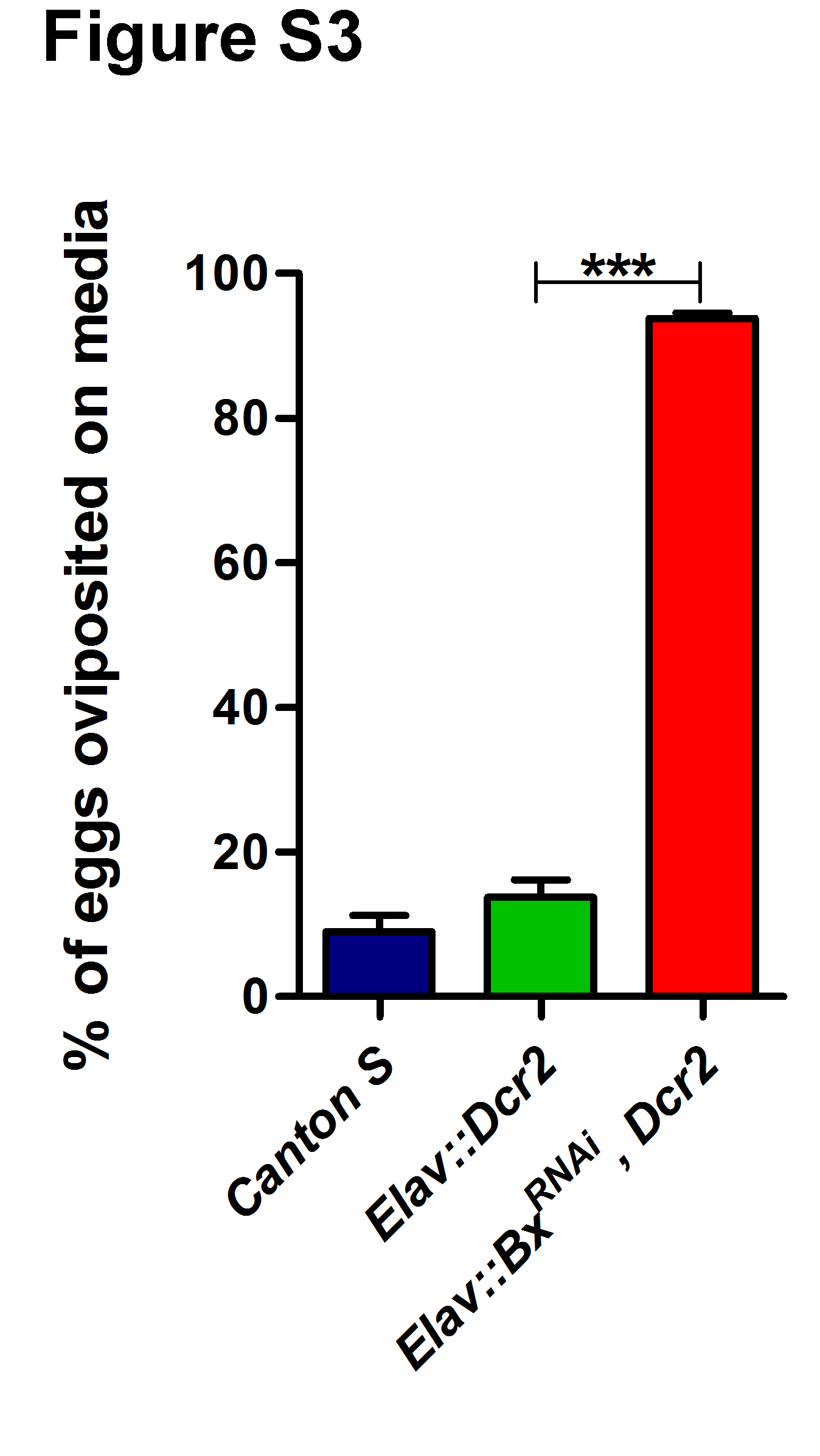

Supplement: Figure S3 — Bx knock down in the neurons show oviposition defect. Knock down of Bx in the neurons leads to defective oviposition where in close to 100% of the eggs was deposited on the surface of the media. (TIF) [file pone.0113003.s003.tif]

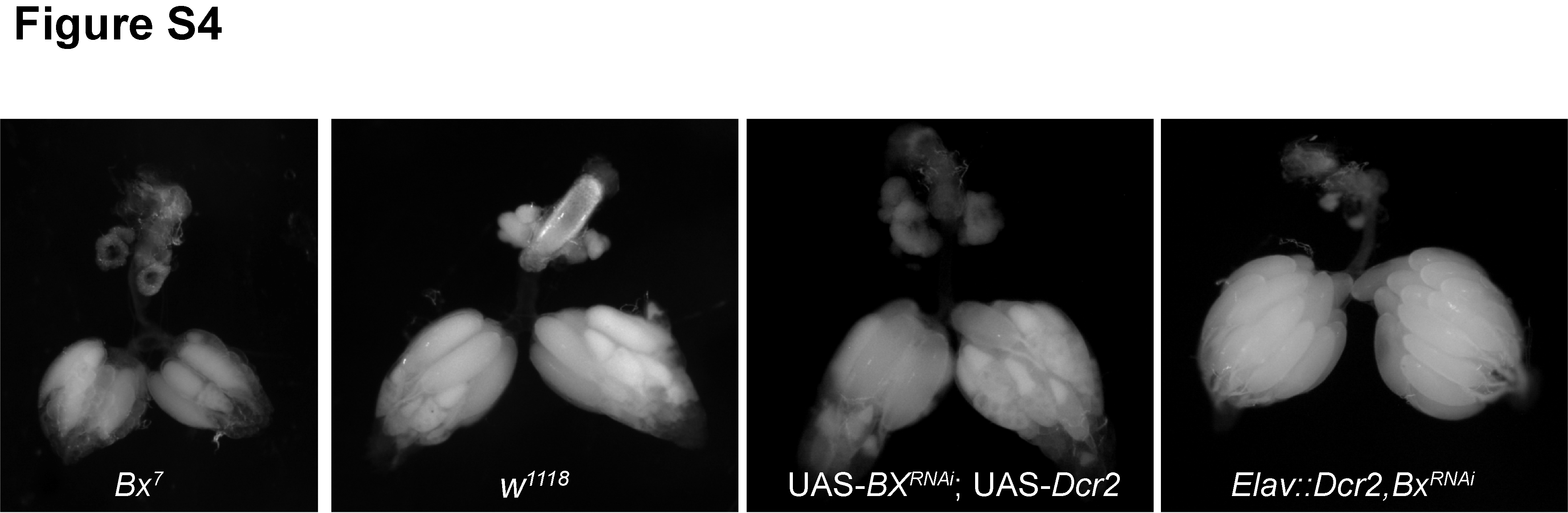

Supplement: Figure S4 — Mature eggs accumulation in Bx7 and the neuronal knock down of Bx females. Bx7 females do not show accumulation of mature eggs in the ovaries unlike those of control ovaries (w1118). However, knock down of Bx in the neurons in females leads to accumulation of mature eggs in the ovaries unlike controls. (TIF) [file pone.0113003.s004.tif]

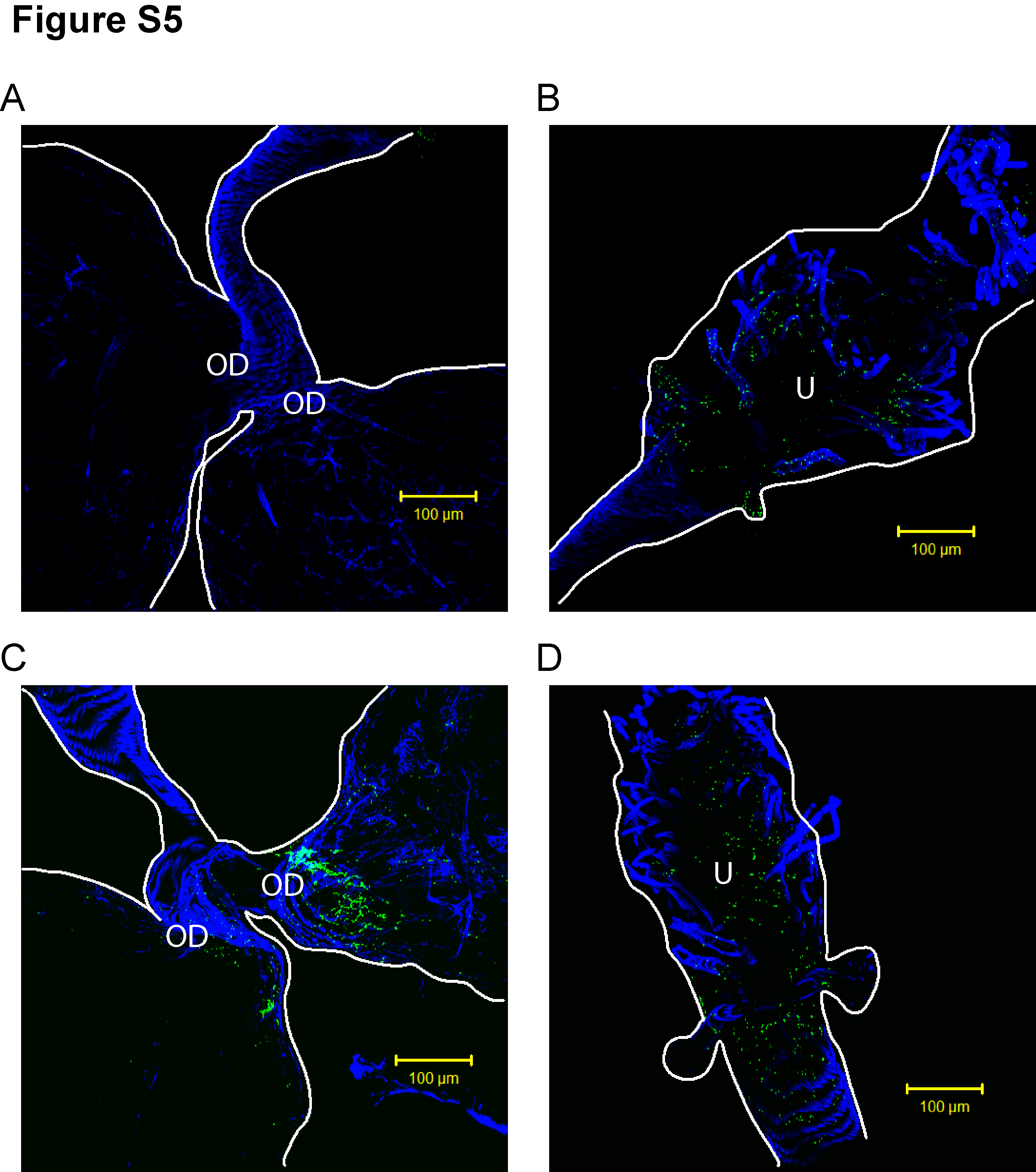

Supplement: Figure S5 — Innervation of VGlut -Gal4 and dTdc2 -Gal4 classes of neurons onto the female reproductive tract muscles. VGlut Gal4 class of neurons showed projections only onto uterine musculature (B, U) but not to the oviduct or common oviduct (A, OD). dTdc2-Gal4 class of neurons showed projections onto the oviduct (C, OD) and uterine musculature (D, U). Blue-Phalloidin-TRITC and Green-Syt-eGFP. (TIF) [file pone.0113003.s005.tif]

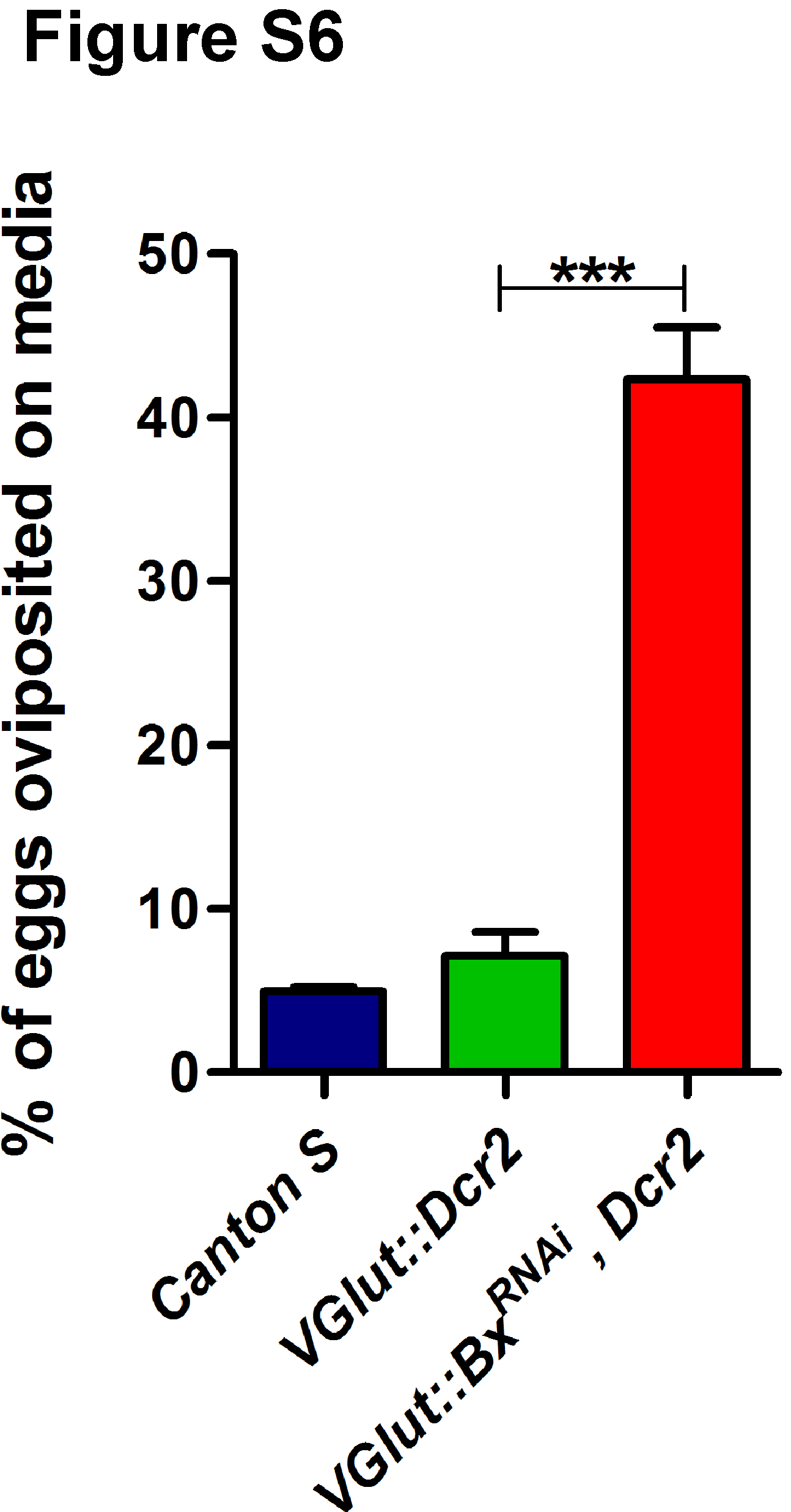

Supplement: Figure S6 — Knock down of Bx in the glutamatergic neurons affects oviposition. Knock down of Bx in the glutamatergic neurons leads to oviposition defect similar to Bx7 mutant females, though to a smaller percentage. (unpaired t-test, ***, p<0.0001). (TIF) [file pone.0113003.s006.tif]

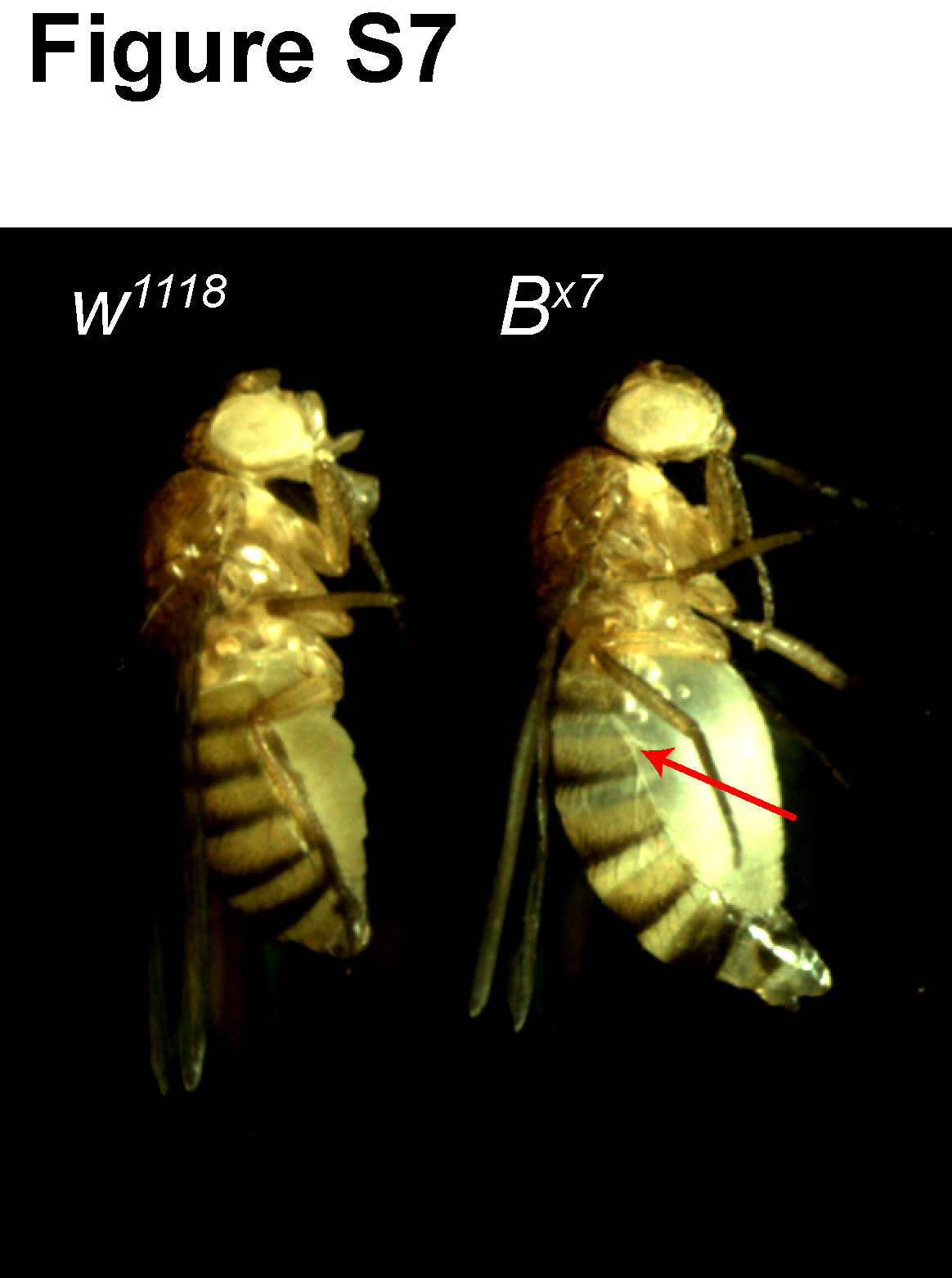

Supplement: Figure S7 — Crop distension in Bx7 mutant females reduces abdominal cavity space. Bx7 mutant females showed increase in the abdominal size due to distension of crop caused due to reduced food movement along the gastric tract. This might lead to inhibition of mature egg accumulation in the Bx mutant ovaries. Red arrow indicates bulged abdomen and crop. (TIF) [file pone.0113003.s007.tif]

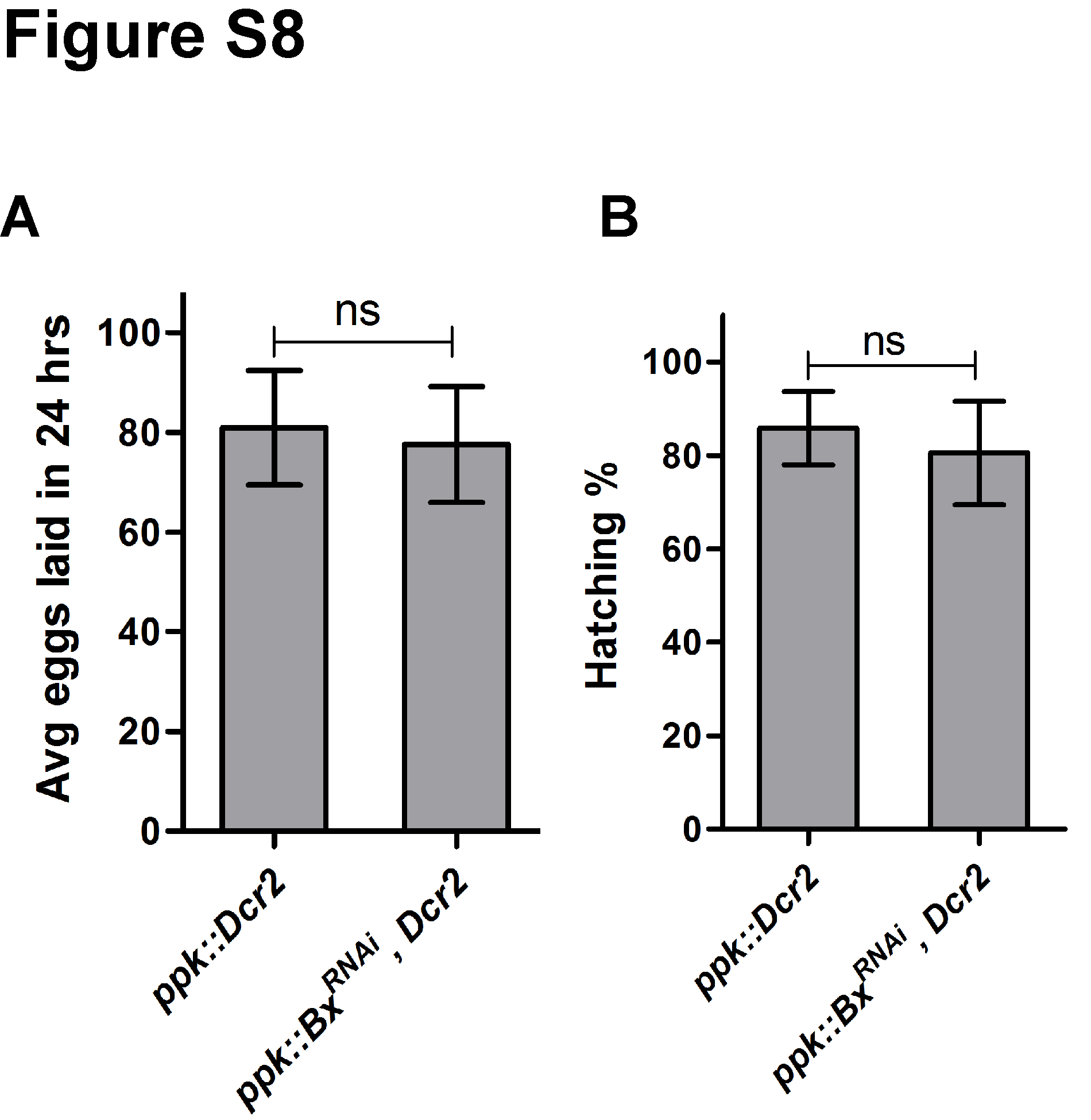

Supplement: Figure S8 — Bx does not regulate female reproduction through the sensory neurons in the female reproductive tract. Bx was knocked down in the sensory neurons in the female reproductive tract with ppk-Gal4. However, this does not reduce either fecundity (A) or fertility (B) of the females. (TIF) [file pone.0113003.s008.tif]
